# Digital approaches to enhancing community engagement in clinical trials

**DOI:** 10.1038/s41746-022-00581-1

**Published:** 2022-03-25

**Authors:** Rayner K. J. Tan, Dan Wu, Suzanne Day, Yang Zhao, Heidi J. Larson, Sean Sylvia, Weiming Tang, Joseph D. Tucker

**Affiliations:** 1 University of North Carolina Project-China, Guangzhou, China; 2 Dermatology Hospital of Southern Medical University, Guangzhou, China; 3 Saw Swee Hock School of Public Health, National University of Singapore, Singapore, Singapore; 4 Clinical Research Department, Faculty of Infectious and Tropical Diseases, London School of Hygiene & Tropical Medicine, London, UK; 5 Department of Medicine, University of North Carolina at Chapel Hill, Chapel Hill, NC USA; 6 School of Social Science, University of Queensland, Brisbane, QLD Australia; 7 Department of Infectious Disease Epidemiology, London School of Hygiene & Tropical Medicine, London, UK; 8 Gillings School of Global Public Health, University of North Carolina at Chapel Hill, Chapel Hill, NC USA

**Keywords:** Clinical trials, Research management

## Abstract

Digital approaches are increasingly common in clinical trial recruitment, retention, analysis, and dissemination. Community engagement processes have contributed to the successful implementation of clinical trials and are crucial in enhancing equity in trials. However, few studies focus on how digital approaches can be implemented to enhance community engagement in clinical trials. This narrative review examines three key areas for digital approaches to deepen community engagement in clinical trials—the use of digital technology for trial processes to decentralize trials, digital crowdsourcing to develop trial components, and digital qualitative research methods. We highlight how digital approaches enhanced community engagement through a greater diversity of participants, and deepened community engagement through the decentralization of research processes. We discuss new possibilities that digital technologies offer for community engagement, and highlight potential strengths, weaknesses, and practical considerations. We argue that strengthening community engagement using a digital approach can enhance equity and improve health outcomes.

## Introduction

Technological advances in the past decades have brought about the rise of digital research methods in health, which have been defined as research that involves the use of the internet, or research that is embedded in online devices^1^. This has also led to the growth of the field of digital health, which the World Health Organization (WHO) defines as the use of digital and mobile technologies to support health system needs. Recognizing the emergence of digitization in both health systems functioning and research, the WHO released the first evidence-based guidelines on digital health in 2019, which discusses research considerations for digital health interventions^2^. This is especially pertinent given the growing trend in connected technologies and digital products^3^. Although much has been written about the digitization of clinical trials in the areas of community recruitment, retention, data collection, and analytic approaches^4,5^, less research has considered how technology can be used to optimize community engagement in clinical trials.

Community engagement has been defined as “the process of working collaboratively with groups of people who are affiliated by geographic proximity, special interests, or similar situations with respect to issues affecting their well-being”^6^. Optimizing the breadth and depth of community engagement goes beyond asking patient representatives about appropriate incentives to participate and other recruitment-focused questions. A continuum of community engagement has been proposed by the United States Department of Health & Human Services, with increasing engagement characterized by community involvement, impact, trust and communication flow spanning from outreach (least engaged), consult, involve, collaborate, and shared leadership (most engaged)^7^.

International organizations like UNAIDS and WHO have underlined the importance of community engagement in clinical trials^8,9^. Community engagement processes have contributed to the successful implementation of trials by helping to identify digital health measures that are relevant to patients and communities, and address barriers to recruitment and implementation of trial components^10–12^. These are especially important given implementation challenges such as mistrust in research, low levels of participation, and insufficient uptake of research findings in communities^13^. Community engagement in research can also be seen as an ethical imperative. Early oral tenofovir trials for HIV pre-exposure prophylaxis among marginalized communities in Cambodia, Cameroon, Nigeria, Malawi, and Thailand drew international attention with concerns over ethical violations in research practices as a result of shortcomings in community engagement^14^.

Given the importance of community engagement in clinical trials, our efforts to digitize research processes in clinical trials should also be reflected in our efforts to strengthen community engagement in such trials. Digital approaches to community engagement are especially important for two reasons. First, the COVID-19 pandemic has led to the transition of many traditionally in-person community engagement activities towards digital adaptations. This trend has accelerated the growth of digital research methods and the implementation of digital health innovations. Second, in the context of digital clinical trials, while technological innovations hold promise in scaling up and improving precision of clinical trials, technological advances alone do not guarantee equitable health outcomes. Many people are inadvertently excluded from digital clinical trial participation, despite being digitally connected, eager, and willing to contribute^15,16^. Rapid digitization without consideration for community engagement processes may therefore result in deepening health inequities.

The purpose of this narrative review is to examine the areas where digital methods have been adopted in clinical trials, and how they have helped enhance community engagement. To identify relevant papers, a search of PubMed was conducted in June 2021 using the following search terms: (digital OR online) AND (community engagement OR equity OR crowdsourcing OR research methods) AND (clinical trial* OR experiment* OR intervention*). Only papers published in English were reviewed, and after reviewing existing studies, digital approaches to enhancing community engagement in trials are discussed. Through our review, we noted that studies utilizing digital approaches that discussed its implications for community engagement or participant equity revolved about the digitization of trial processes, the use of online crowdsourcing methods, as well as online qualitative research methods in trial contexts. We selected these three areas of focus as well because of their importance within WHO frameworks, relevance to digital community engagement, and potential impact on health equity^17,18^. Consequently, the three areas of focus for our review include (i) the use of digital technologies to implement trial components, (ii) digital crowdsourcing to develop trial components, and (iii) the use of digital qualitative research methods for community engagement.

## Digital technology for trial processes to decentralize trials

Digitization of clinical trial processes can enhance community engagement by improving representation of participants, generalizability of results, and equity of outcomes through the decentralization of trial processes. Digitization of clinical trials involves the use of technology for the implementation of trial processes, which include eligibility assessment and consent in recruitment, allocation, intervention, outcome assessment, and dissemination of findings^4,19^. Such digitization processes are becoming increasingly common as the COVID-19 pandemic has driven many clinical trials to embrace online components in whole or in part as a result of policies that have limited in-person interactions, and encouraged digital assessments within clinical trials. Some in-person interventions have been entirely transitioned into online formats in response to COVID-19^20^.

Technology can be employed in clinical trial implementation procedures in ways that will enhance community engagement and equity. First, digital clinical trials have the potential to be more inclusive in terms of the communities that are engaged, as well as allow for decentralization of trial processes and reduce costs. Specifically, digital recruitment of participants may include marginalized people who live in rural areas who may have greater difficulty travelling to clinical trial sites at academic medical centers, which allow for a broadening of recruitment criteria to include participants regardless of where they reside or work^4^. For example, a crowdsourcing contest that solicited online submissions in Eswatini saw substantial participation from participants in rural areas by combining online participation with offline promotional methods^21^. In the United States, a similar crowdsourcing approach to garner online community feedback on HIV clinical trial processes saw participation from more women, more racial and ethnic minorities, and more low-income individuals compared to a traditional community advisory board (CAB) approach^22^. Second, technological implementations also allow for capturing data from digital interventions that are often decentralized and not attached to specific clinical settings, including objective data on behavior or biological measurements, reducing the need for participants to travel to clinical settings for participation. For example, a randomized trial evaluating a mobile phone application detecting potential arrhythmias enrolled 419,297 participants who were not required to attend a clinic^23^. This included participation from typically underrepresented groups in clinical trial studies^24^, such as racial or ethnic minorities (32% were non-white) and those who are older (10% were 55–64 years old; 5.9% were 65 years old and above). Furthermore, the use of wearables like activity trackers and smartwatches has shown promise in the fight against COVID-19^25,26^, and may pave the way for more inclusive participation if made available to a diverse population. Finally, digital clinical trials may reduce costs associated with recruitment and retention compared to conventional in-person trial designs^4^, and therefore costs for participants as well.

Despite these benefits for community engagement, there are also weaknesses associated with digital technologies when implementing trial components or processes. First, the persistence of a digital divide excludes populations from participating in such digital activities^27^. Often older people, people with disabilities, and financially disadvantaged people are digitally excluded. Second, self-reporting is a source of potential bias in online surveys that may be used for assessments in clinical trials, as opposed to surveyor-administered surveys in clinic settings. Smartphone photo-verification of some outcomes (e.g., self-test completion) can decrease reliance on self-report^28^. Third, in trials that evaluate social media messages, contamination between study arms is a potential problem. Such messages could be shared online, exposing participants in the control arm to the intervention^29^. Methods to decrease contamination include limiting exposure to intervention messages in the setting of closed private groups, sending messages that cannot be saved or shared, and limited intervention exposure to in-person settings. In addition, verification of human participants is important and can be achieved through simple CAPTCHA (Completely Automated Public Turing test to tell Computers and Humans Apart) widgets. Finally, there is at present few mandatory reporting standards for the use of digital health-specific research checklists, in spite of the availability of one research checklist for digital survey studies^30^, and a CONSORT eHealth checklist for digital health interventions^31^. Nevertheless, efforts are being made to improve standards around the design and reporting of digital trials, such as The Clinical Trials Transformation Initiative’s digital health trials program focused on decentralized clinical trials, novel endpoints, engaging patients, and technology^32^.

## Digital crowdsourcing to develop trial components

Digital crowdsourcing processes present opportunities to enhance diversity in representation not just among trial participants, but for community input and engagement processes in the design of clinical trials. Crowdsourcing activities are increasingly used in digital health research^33^, and typically involves a group of diverse individuals contributing to solving a health problem and then sharing identified solutions with key stakeholders^34^. This in turn may help tailor health programs to the needs of end users to improve acceptance, and limit the homogeneity of interventions developed using a top-down approach. Crowdsourcing is often implemented using digital technologies, and as such may provide an important opportunity to fill the gap in community engagement in clinical trials. This may be achieved specifically through open calls for clinical trial intervention materials, as well as the use of digital hackathons to further develop crowdsourced ideas.

Open calls and hackathons are crowdsourcing activities that can be implemented on digital platforms and used for participatory development of clinical trial components^35^. Evidence suggests that crowdsourced interventions are potentially superior to conventional approaches, in improving health outcomes. Examples include the superiority of crowdsourced methods in developing sexual health intervention material by the general public vis-à-vis those developed by experts^35,36^. Promotional events can be conducted online using websites, email dissemination, social media platforms, such as Facebook, WeChat^37^, or TikTok^38^. For example, TikTok users participated in a crowdsourcing open call to identify a user-friendly medicine bottle design for people living with Parkinson’s disease^38^.

In addition to open calls, digital hackathons are another form of crowdsourcing that can further increase community engagement in clinical trials^39^. Digital hackathons typically involve an open call for submissions, which lead up to intensive activities that bring people together via online platforms to complete a specific task within several days^35^. Digital hackathons can foster interdisciplinary collaboration, and lead to rapid innovations for clinical trials to tackle health challenges. For example, digital hackathons have been implemented in response to the COVID-19 pandemic to crowdsource challenges faced by individuals from public, private, and non-governmental organizations in the pandemic, and to generate rapid solutions to address urgent and unmet needs that have arose^39–41^. Apart from generating innovative ideas for evaluation in clinical trials, hackathons have been used to refine and determine optimal implementation strategies for trials^35^. Previous research has shown that hackathons can take ideas solicited via an open call forward and optimize public health campaign messages, which are proven non-inferior to conventional expert driven interventions as part of a clinical trial^42,43^.

Crowdsourcing techniques that comprise digital channels for participation can also complement existing CAB arrangements to broaden stakeholder feedback. CABs are typically composed of individual stakeholders who reflect the community of interest, and serve as a source of leadership to guide research activities and trial procedures^44^. The US National Institutes of Mental Health first required CABs to enhance community engagement in HIV research in 1987^45^. A study comparing levels of engagement between a traditional CAB and crowdsourced wisdom found that crowdsourcing may deepen engagement and inclusion of community voices, especially among unemployed individuals and people with disabilities^22^.

Crowdsourcing activities have several advantages. By engaging a group of people with diverse backgrounds, crowdsourcing may have higher potential for innovation due to multi-sectoral, interdisciplinary contributions to creating culturally appropriate solutions that may inform the design and implementation of clinical trials^35,36^. Digital approaches to crowdsourcing can further increase the scale of the event, amplify the reach of audience and the diversity of cultural backgrounds of participants by breaking physical barriers, especially where access to in-person events may serve as an even larger barrier to participation among such marginalized groups, rather than the need for technical assistance^22^. To mitigate limited participation from marginalized subgroups as a result of individual and structural barriers, strategies for engagement need to be sensitive to availability and distribution of technology among participants. For example, framing the topic as broadly relevant to key communities and allowing the use of audio and video clips, images, and texts, or other artistic formats may enable people to create a submission in their own way, including those with disabilities^46^. Flexibility in submission channels can also be considered such as allowing participants who encounter difficulties in submitting via a survey link to send their entries via social media apps or emails^47^.

On the other hand, while digital crowdsourcing activities have successfully been implemented in low- and middle-income countries (LMICs)^48^, limited access to technology may be a barrier to participation. It is therefore important for researchers to examine the digital ecosystem and structures of a particular context before implementing digital crowdsourcing approaches to avoid such selection biases that contribute to the digital divides. For example, past crowdsourcing activities in LMICs have considered barriers to participation such as internet bandwidth issues, and have adopted asynchronous digital technologies and social media apps, such as Facebook, WhatsApp, and WeChat, can be leveraged to organize and promote the crowdsourcing activities^21^. Furthermore, digital crowdsourcing methods can be complemented by CABs or co-creation group efforts to enhance community engagement, given that CAB members may be more familiar with trial processes and are better positioned to identify broader institutional or systemic concerns inherent to trials^22^.

## Digital qualitative methods to amplify and diversify participant voices

Digital qualitative methods can be used in clinical trial processes to enhance representation in formative and process evaluation research for clinical trials, as well as lend greater ecological validity to research findings due to the decentralized nature of such approaches. Past work has established how such methods are well-suited to address important questions that may arise in formative clinical trial research and in the implementation sciences, such as exploring the multi-faceted contexts where implementation takes place, the processes and steps involved in implementation, as well as the effectiveness of implementation strategies^49^. In the context of formative research, studies have shown that formative qualitative research can help assess the usability, feasibility, acceptance, and impact of potential digital health interventions^50^, and for the reasons mentioned above, can help improve representation and ecological validity for the implementation of clinical trial components. In the context of process evaluation, past studies using digital storytelling have found such techniques are useful to elicit both visual and textual representations of lived experiences and the contexts in which participants are embedded in^51,52^, which are useful for understanding how interventions in clinical trials may work, for whom, and under what contexts^53^.

While COVID-19 has disrupted the ways such methods have traditionally been conducted in-person due to movement control measures and the risks of in-person participation, it has also given us the opportunity to focus and expand on existing digital qualitative methods. This includes data collection by telephone, text^54^, and videoconference^55^. Digital forms of focus group discussions (FGDs) have also emerged, starting with the use of emails, online forums and message boards^56^, and later, virtual discussion rooms which bear a strong resemblance to traditional focus groups in their synchronous nature but also rely on text-based nonverbal communication and discussion^57^. More recently, scholars have also used teleconferencing software and chat apps such as WhatsApp to conduct both asynchronous and synchronous FGDs^58^. Traditional participant observation methods have also evolved towards innovative approaches such as cyber-ethnography, as well as participant-led methods like photovoice, that provide promising advances in social methodology and inquiry^59,60^.

Community engagement using digital qualitative methods have several benefits for clinical trials. First, digital platforms may help deepen community engagement by creating safer environments or spaces for community engagement, and foster more authentic feedback from community members. For example, online chat-based FGDs may facilitate the sharing of sensitive information from, or disagreements among participants due to the absence of nonverbal cues, as such cues may inform perceived power differences among participants^61,62^. Chat-based mobile phone apps also offer high ecological validity due to their embeddedness in individuals’ everyday lives^63^. The anonymous nature of online spaces may increase trust in cyber-ethnography compared to other engagement methods because of the lower risk of self-disclosure and resulting safe space^64^.

Second, digital qualitative methods also offer logistical advantages for inclusivity, thus deepening engagement through greater representation and diversity among participants. For example, online FGDs can be more inclusive in terms of recruiting a diverse range of participants who may face access issues when getting to physical locations^65^. The use of digital qualitative methods may also save time and costs for researchers who do not have to pay for in-person venue rental fees, transportation, and transcription in some cases^58,66^. Digital qualitative methods also decrease COVID-19 risks associated with conventional in-person qualitative methods^67^.

Nevertheless, there are several drawbacks of community engagement using digital qualitative methods, which may have implications for the implementation of clinical trials. With regard to the depth of data generated, researchers have found that online chat-based interviews and FGDs tended to generate lower word counts, shorter responses, and provided less detail or richness^57,61,68,69^. The lag time between responses, as well as the lack of an ability to sense emotions such as sarcasm or the use of metaphor may lead to ambiguity in interaction^70^. Online chat-based FGDs also tended to have fewer group interactions and lower responsiveness to facilitators’ questions and probes^57,62,68^. While videoconferencing software has allowed for an approximation of in-person interviews and FGDs, researchers may encounter difficulties with disruptive environments, the loss of concentration, as well as a lack of nonverbal cues and body language^55,71^. Ethical issues may also arise with methods such as cyber-ethnography, where informed consent may be difficult to obtain from participants due to the often unobtrusive and covert nature of cyber-ethnographic approaches^72^.

## Strengthening equity through digital community engagement

We present a summary of how the three digital approaches highlighted above can be applied throughout the life course of clinical trials (Table 1). Enhancing community engagement through digital approaches can support equitable processes and outcomes in clinical trials. Our review highlights how digital technologies not only provide an alternative means of engaging participants in trials but also provide unique information to enhance clinical trials. These generally include the ability to engage participants and experts in multiple settings, regardless of geography and nationality through the use of digital technologies, reduced costs associated with the use of such online or digital components, and opportunities to democratize trial design processes.

**Table 1:** Summary of digital approaches to community engagement and its application in the life course of clinical trials.

Digital approaches	Digital trial processes	Crowdsourcing	Qualitative methods
Developing trial components	–	Open calls and hackathons	–
Formative research	–	Open calls and hackathons	Formative qualitative research
Participant recruitment and retention	Remote recruitment of participants	Crowdsourced participant recruitment	Process evaluation and implementation science approaches
Implementation	Decentralized trial components	Crowdsourcing to complement community advisory boards	Process evaluation and implementation science approaches

While digital approaches employed in clinical trials have a potentially wider reach than in-person trials, they can exacerbate health inequities if care is not taken to ensure that the inclusive potential of such approaches are realized. Mobile phone subscriptions may be rising globally, including LMICs; however, mobile network coverage has been found to be lower and more expensive in the least developed countries of the world^73^. And while mobile phone subscriptions are rising rapidly across the world, the same cannot be said about access to the internet, where mobile and fixed broadband subscriptions per capita are lagging behind in developing economies, compared to the rest of the world^27,74^. The lack of consideration in the design of interventions and commercial products for those with limited access to broadband or internet bandwidth may also exacerbate such inequities^75^. Furthermore, beyond the availability of technology, past studies evaluating the effectiveness of digital health services in a given context have shown that social determinants of health including socioeconomic status, demographic attributes, and levels of health literacy impact one’s access to digital health interventions and thus engagement in clinical trials^76^.

**Fig. 1:**
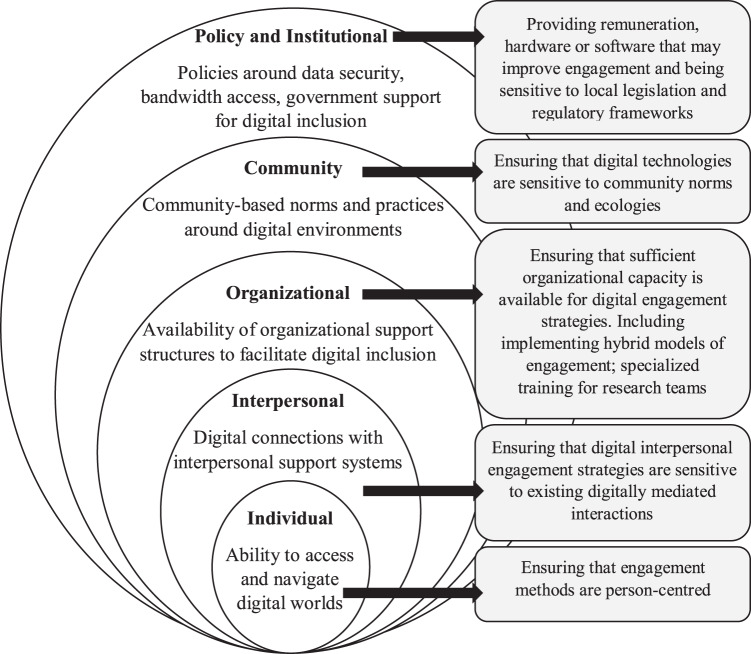
Revised socio-ecological model and recommendations for digital inclusion. Revised socio-ecological model providing considerations and potential solutions for digital inclusion at the individual, interpersonal, organizational, community, and policy levels.

Digital access has become a social determinant of health with increasing salience amidst the COVID-19 pandemic, as health information and interventions are increasingly being rolled out and implemented online, leaving the most vulnerable behind^77^. We present an adapted socio-ecological model (Fig. 1) which underscores important considerations at the individual, interpersonal, organizational, community, and policy levels for digital engagement strategies that clinical trials should consider. We also present several recommendations for clinical trials to enhance community engagement in these areas, including person-centered strategies at the individual level, sensitivities to digitally mediated interactions at the interpersonal level, ensuring organizational capacity through specialized training or allowing for flexible hybrid models of engagement at the organizational level, sensitivities to community norms and ecologies at the community level, and ensuring availability of resources and alignment with legal and regulatory frameworks at the policy and institutional level.

## Addressing participant privacy and regulatory barriers

Patient and participant privacy remain key concerns with the digital approaches in research and community engagement^78^. As digital approaches to community engagement hold promise in reaching diverse participants who are typically underrepresented in clinical trial research, privacy protections need to shift in tandem with potentially heightened vulnerabilities that such communities face. For example, individuals who are socioeconomically disadvantaged may face obstacles negotiating digital privacy and confidentiality and require tailored frameworks to uphold data governance principles and mitigate risks to privacy^79^. Furthermore, privacy concerns may arise from digital crowdsourcing techniques and qualitative approaches, as open calls rely on the use of online systems as well as principles of open access to information and materials. These may pose ethical risks if privacy safeguards are not considered^80^. Potential safeguards include limiting the identifying information collected as part of digital engagement and not requiring the use of real names when taking part in studies, allowing participants to opt out of specific questions and still join a study or engagement project, and co-creating study protocols with communities in considering the ethics and study design to minimize potential risks. Risk mitigation strategies for crowdsourcing contests and innovation challenges have also been detailed elsewhere^81^.

Regulatory hurdles may also need to be addressed to facilitate community engagement through digital approaches. Specifically, existing regulatory frameworks such as those that govern the use of digital platforms, medical devices or therapeutic approaches may not be favorable for novel approaches in digital health, therefore posing barriers to innovation and adoption^82^. Regulatory innovations, including regular adjustments to frameworks or regulatory flexibilities in emergency situations^83^, are required by governance structures to ensure that digital innovations suggested in this article can be effectively implemented. Such regulatory innovations have been effectively implemented in the COVID-19 pandemic without compromising on patient safety or product quality^83–85^. Additionally, regulatory frameworks that govern research practices or community engagement in trials may also require further review to ensure that research protocols are aligned with practices that uphold patient privacy and safety in the context of novel digital approaches^86,87^.

## Discussion

In conclusion, digital approaches can help to promote health equity in digital clinical trials in several ways (Table 2). In Table 2, we also provide examples of each digital approach and describe how they had enhanced community engagement.

**Table 2:** Digital community engagement methods and its role in enhancing equity in digital trials.

Digital approaches	Contributions to clinical trials	Pathways to equity	Examples and case studies
Digital technology for trial processes to decentralize trials	• Digitizing trial implementation components	• Enhance representation of participants, generalizability of results, and equity of outcomes in digital trials	• Online surveys, online implementation of trial components. [See the Apple Health study in the United States described in the main text by Perez et al.^23^].
Digital crowdsourcing to develop trial components	• Developing and introducing crowdsourced interventions • Refining and building on crowdsourced ideas to enhance their effectiveness • Digitizing traditional community engagement methods (e.g., community advisory boards)	• Enhancing diversity and representation in the generation and development of ideas that inform digital trials • Providing flexibility in participation channels for crowdsourced open calls through varying channels enhance access in spite of varying levels of digital technologies available to individuals • Providing opportunities to engage in virtual CABs to enhance attendance and access to CAB meetings, and enhancing inclusion by crowdsourcing perspectives to enhance complement that of conventional CABs	• Open calls for logos, posters, videos, and other intervention material for evaluation and health promotion in clinical trials [See the study by Tang et al. on the use of digital crowdsourcing methods combined with an in-person hackathon to develop a crowdsourced intervention in China^43^]. • Hackathons for winning entries in open calls to further develop and field test ideas for eventual implementation [See the case study of an online hackathon to combat the COVID-19 pandemic in Germany, published by Braune et al.^39^]. • Running concurrent crowdsourced open calls to complement CABs in a trial [See case study published by Day and colleagues of a comparison between, and unique contributions of crowdsourcing and CAB to feedback on a phase 1 HIV antibody trial in the United States^22^].
Digital qualitative methods to amplify and diversify participant voices	• Formative qualitative research • Process evaluation and implementation science strategies	• Enhancing access to participate in formative qualitative research among participants who may face difficulties in accessing in-person facilities • Enhancing ecological validity through the purposive selection of specific digital qualitative methods that are sensitive to the availability of technologies and distribution of digital access in a given setting	• Use of teleconferencing software to conduct in-depth interviews with trial participants [See example of an article published by Oliffe et al. on the benefits and concessions of Zoom interviews among Australian and Canadian men in the context of intimate partner relationship breakdown amid COVID-19^88^]. • Use of WhatsApp for conducting focus group discussions with participants, allowing for both synchronous and asynchronous group discussions [See publication by Colom on the use of WhatsApp focus group discussions among young activists in Kenya on the benefits of such digital qualitative approaches^63^.]

This narrative review highlighted potential strengths and weaknesses across three broad digital engagement strategies. However, a digital divide and barriers owing to participant privacy and regulatory hurdles remain, which threaten the implementation of such digital approaches. Nevertheless, research has demonstrated that digital approaches can enhance equity in clinical trials and be modified in ways that are sensitive to local access to digital technologies, which may be more heterogeneous in LMICs where access to affordable mobile data and broadband are limited.

This narrative review also provides trial researchers and implementation scientists strategies to deepen community engagement in clinical trials. For researchers engaged in clinical trials that already adopt community engagement processes in their work, digital approaches provide ways to enhance equity and more broadly engage communities. For clinical trial researchers who have not yet adopted community engagement techniques, digital methods for engaging communities should be considered. Regulators and expert committees should also consider providing recommendations on the benefits and limitations of such digital approaches to engaging communities in clinical trial research as future considerations in clinical trial frameworks.

### Reporting summary

Further information on research design is available in the Nature Research Reporting Summary linked to this article.

## Supplementary information

Reporting Summary

## Data Availability

All data generated or analyzed during this study are included in this published article. All aggregate data collected for this Review are available from the corresponding author on reasonable request.
